# Annotations

**Published:** 1905-02-25

**Authors:** 


### ANNOTATIONS.

### A Practical Protest against the Voting System.

A feature of the remarkable will of the late Mr. Frederic David Mocatta, who has left large sums of money to various hospitals and other charities, is that the testator signifies the abandonment of a design which he originally entertained. It was his intention to make bequests to many institutions, such as the Hospital for Incurables at Putney and the Asylum for Idiots at Earlswood, but after much consideration and experience, he became convinced that the system of electing those to be benefited by the institution by means of the votes of subscribers instead of according to the merits of each case, as ascertained by careful investigation by the committee of the several institutions, is "radically unsound and wrong, and frustrates the best aims of charity." Accordingly, he considered it to be his duty not to leave legacies to any institution which adopts that system, though he bequeathed £1,000 to the Jews' Hospital and Orphan Asylum on the express condition that the authorities abolish voting within 10 years of his death. Probably he had satisfied himself that it was useless to make the other bequests he had contemplated on the same condition. We do not anticipate that the institutions in question will be influenced by the publication of Mr. Mocatta's conviction, based as it was upon ripe knowledge and keen judgment. Of course, they regret the loss of legacies which are always acceptable. But old customs die hard, and there is no doubt that some boards of management still honestly labour under the delusion that the system condemned by Mr. Mocatta is the best for the institutions, because people like having value for their money, and every subscription purchases a vote, and the fairest for the candidates, because the election of patients depends upon so many persons that neither undue influence nor prejudice can be successfully exerted. The liberal support given to the great hospitals, to which admission is absolutely free, is a conclusive proof of the fallacy of the one contention, while as to the other, which is purely hypothetical, the answer is that many most deserving applicants are unsuccessful because they are penniless and friendless.

### "In Corpore Sano."

However defective in the eyes of educational experts the British ideal of scholastic training in our public schools and universities may appear to be, it cannot be denied that the culture of athletics and outdoor sports in these institutions has through many generations produced men competent to play a leading part in the affairs of the world. The higher reaches of scholarship may not have been generally attained, but the vigorous qualities of robust manliness have certainly been encouraged. The value of this result as a national asset is largely realised, and in a recent article by Dr. Macnamara, M.P., proposals are advanced to extend it in such a manner as to include not only the leisured, but also the middle and working classes. The suggestion is that not only shall adequate drill be continued as part and parcel of school life and discipline, but that after leaving school lads shall be compelled to give at least two hours on two nights a week to physical training under State auspices. Such training would include gymnastic exercises and drill needed for formations and combined movements as well as experience in the use of a rifle. It is urged that the adoption of such a proposal would obviate the necessity for conscription, but its special appeal lies in its value as a means for improving the physical development of the citizen. Though some exaggeration has been introduced into various descriptions of the depreciation of the national physique it cannot be doubted that the means for improving bodily development have not kept pace with the increased opportunities for mental training. On this point the medical profession has special and expert knowledge and there rests with its members a corresponding measure of responsibility. Hence we think that there will be a general inclination in the profession to agree with the proposals above described and we venture to suggest that this should lead to active support. If this were afforded on a large scale there is little doubt that a movement carrying both individual and national benefits would result.

### Fraudulent Advertisements.

The melancholy collapse of the so-called "Pension Fund for Widows," which was announced by a certain firm of tea-dealers, is an event calculated to direct serious attention to a blot in our system of public education—a blot which was long ago described by Faraday. There is no education of the judgment, and one consequence is that the increasing number of people who are able to read serves only to increase the number who are ready to be the dupes of advertisements. Any statement which is displayed on highly-coloured posters, however improbable or untrue, is practically certain to be so far believed by large numbers of people as to induce them to spend their often hardly-earned money in commodities which are either worthless or at least not worth the prices demanded for them. Systematic robbery of the poor is carried on in this way chiefly by the descriptions of various preparations which are asserted to be of high nutritive value, and which, too often, take the place of wholesome food. It has often been found, on trial, that the law was quite capable of dealing with new varieties of fraud if only its provisions were fairly enforced; and we cannot but think in relation to many of the advertisements of food preparations that a prosecution for obtaining money under false pretences might be successfully conducted. If no conviction under the statutes dealing with this offence could be obtained, the evidence adduced would surely be sufficient to convince the public of the necessity for an amendment of the law. As regards the wage-earning classes, we believe the question to be one of urgent importance in relation to the feeding of children as well as of adults; the combination of a power to read falsehoods with a complete ignorance of cookery being the bane of large numbers of mothers of the working-classes. For victims of this description we have only pity; and, if in other cases the same sentiment would be out of place, it is none the less to be regretted that successful dishonesty should be permitted to flourish unchecked.

